# Programmed cell death 11 modulates but not entirely relies on p53-HDM2 loop to facilitate G2/M transition in colorectal cancer cells

**DOI:** 10.1038/s41389-023-00501-2

**Published:** 2023-12-07

**Authors:** Li Ding, Yujie Xu, Lin Xu, Chenhong Zhao, Zhiping Zhang, Jie Zhang, Kai Liao, Yuerou Chen, Jingwen Li, Xinyu Mei, Xinyue Zhang

**Affiliations:** 1 College of Bioscience and Biotechnology, Yangzhou University, Yangzhou, 225009 Jiangsu China; 2 Joint International Research Laboratory of Agriculture & Agri-Product Safety, the Ministry of Education of China, Yangzhou University, Yangzhou, 225009 Jiangsu China; 3 Key Laboratory of Prevention and Control of Biological Hazard Factors (Animal Origin) for Agrifood Safety and Quality, the Ministry of Agriculture of China, Yangzhou University (26116120), Yangzhou, 225009 Jiangsu China; 4 Jiangsu Co-innovation Center for Prevention and Control of Important Animal Infectious Diseases and Zoonoses, Yangzhou, 225009 Jiangsu China

**Keywords:** Cell growth, Oncogenes, Ubiquitylation

## Abstract

We previously described a nucleolar protein RSL1D1 but distributed throughout the nucleus in HCT116 colorectal cancer (CRC) cells to facilitate G1/S transition by inhibiting p53 signaling. Here, we found another nucleolar protein, programmed cell death 11 (PDCD11), also with an “Extra-nucleolar” localization in CRC cells but to regulate G2/M checkpoint. This protein directly interacts with p53 and HDM2 in the nucleoplasm, thereby recruiting p53 to HDM2 for ubiquitination and degradation. The ensuing downregulation of p53 increases the CDK1 level to help the cells pass G2/M checkpoint. Upon DNA damage stress, PDCD11 gains the power to upregulate CDK1 independently of p53. Beyond these, PDCD11 also upregulates CDC25C in a p53-independent manner to dephosphorylate CDK1 to facilitate G2/M transition. Downregulation of PDCD11 greatly reduced cancer cell growth in vitro and in vivo, additionally sensitized cells to DNA damage signals, highlighting that PDCD11 is a crucial driving factor of CRC and a potential target for cancer treatment.

## Introduction

Numerous studies reveal the close relationship between nucleolar proteins and p53. It is generally recognized that nucleolar proteins are translocated from the nucleolus to the nucleoplasm in response to nucleolar stress, thereby interacting with and inactivating HDM2, a primary negative regulator of p53, to stabilize p53 protein [1–5]. However, our previous study clarifies a reversed function of nucleolar protein RSL1D1 to increase HDM2-mediated p53 ubiquitination in a cell-type-specific manner [6]. Other than its nucleolus-specific localization in normal cells or other types of cancer cells [5, 7, 8], RSL1D1 is overexpressed and colocalized with p53 and HDM2 throughout the nucleus of HCT116 colorectal cancer (CRC) cells [6]. This facilitates RSL1D1 to directly interact with p53 and HDM2 under non-stress condition, thereby recruiting p53 to HDM2 for degradation and thus inhibiting the p53-p21/PUMA signaling. Accordingly, we defined RSL1D1 as an oncoprotein to help CRC cells escape from apoptosis and G1/S cell-cycle checkpoint. Since rapid growth of cancer cells were majorly attributed to the abnormal activity of cell-cycle machinery and less controlled G1/S and G2/M transition [9], we wondered whether some other nucleolar proteins downregulate p53 in CRC cells to help pass G2/M checkpoint, thereby cooperating with RSL1D1 to promote CRC progression.

By interrogating The Cancer Genome Atlas (TCGA) [10] and the Genotype-Tissue Expression (GTEx) [11] databases, we found another nucleolar protein, programmed cell death 11 (PDCD11), overexpressed in colorectal tumors and highly related to CRC progression. PDCD11 (Gene ID: 22984) [12], also known as ALG-4 [13], RRP5 [14], or NFBP [15], is located on human chromosome 10q24.33, contains 37 exons, and encodes a translational product with 1871 amino acids. This protein possesses a series of S1 RNA-binding domains in the N-terminus with approximately 1,400 amino acids, followed by a tetratricopeptide repeat (TPR) domain in the C-terminus [16]. Through S1 N-terminal domains, PDCD11 interacts with pre-rRNA to coordinate rRNA processing and assembly in the nucleolus [14, 17–19]. It also binds to Exonuclease-1 and upregulates Fas to induce cell apoptosis [13, 20]. Recently, PDCD11 was proved to interact with NF-κB subunits to suppress the expression of inflammatory cytokines and activate the TGFβ1 pathway, thereby modulating microglia differentiation during zebrafish development [21]. Interestingly, the study also shows that PDCD11 deficiency hyperactivates p53 target genes in zebrafish embryo, suggesting that this protein might adversely affect p53-related pathways. However, whether and how PDCD11 directly regulates p53 is still unclear.

To address this question, we performed a series of investigations in the present study. Our data demonstrate that, similar to RSL1D1, high level of PDCD11 is another key factor to negatively regulate p53 in CRC. This protein is overexpressed and not confined to the nucleolus of CRC cells, which facilitates it to colocalize with p53 and HDM2 under normal condition. Through direct interaction in the nucleoplasm, PDCD11 recruits p53 to HDM2 for ubiquitination, leading to a decreased p53 level. Other than the facilitation of G1/S transition by RSL1D1 in HCT116 cells [6], PDCD11 upregulates CDK1 (also known as CDC2) in a p53-dependent manner, also upregulates CDC25C expression to dephosphorylate CDK1 in a p53-independent manner, thereby accelerating G2/M transition and cell proliferation under normal condition. More than that, PDCD11 owns a pro-survival function to resist p53-independent G2/M arrest and p53-dependent apoptosis under DNA damage stress (DDS). We concluded that PDCD11 is an “Extra-nucleolar” oncoprotein to promote cancer cell growth and survival by regulation of G2/M checkpoint and DNA damage signaling, highlighting a potential novel target for treating CRC.

## Results

### PDCD11 acts as an oncoprotein to accelerate colorectal cancer progression

**Fig. 1:**
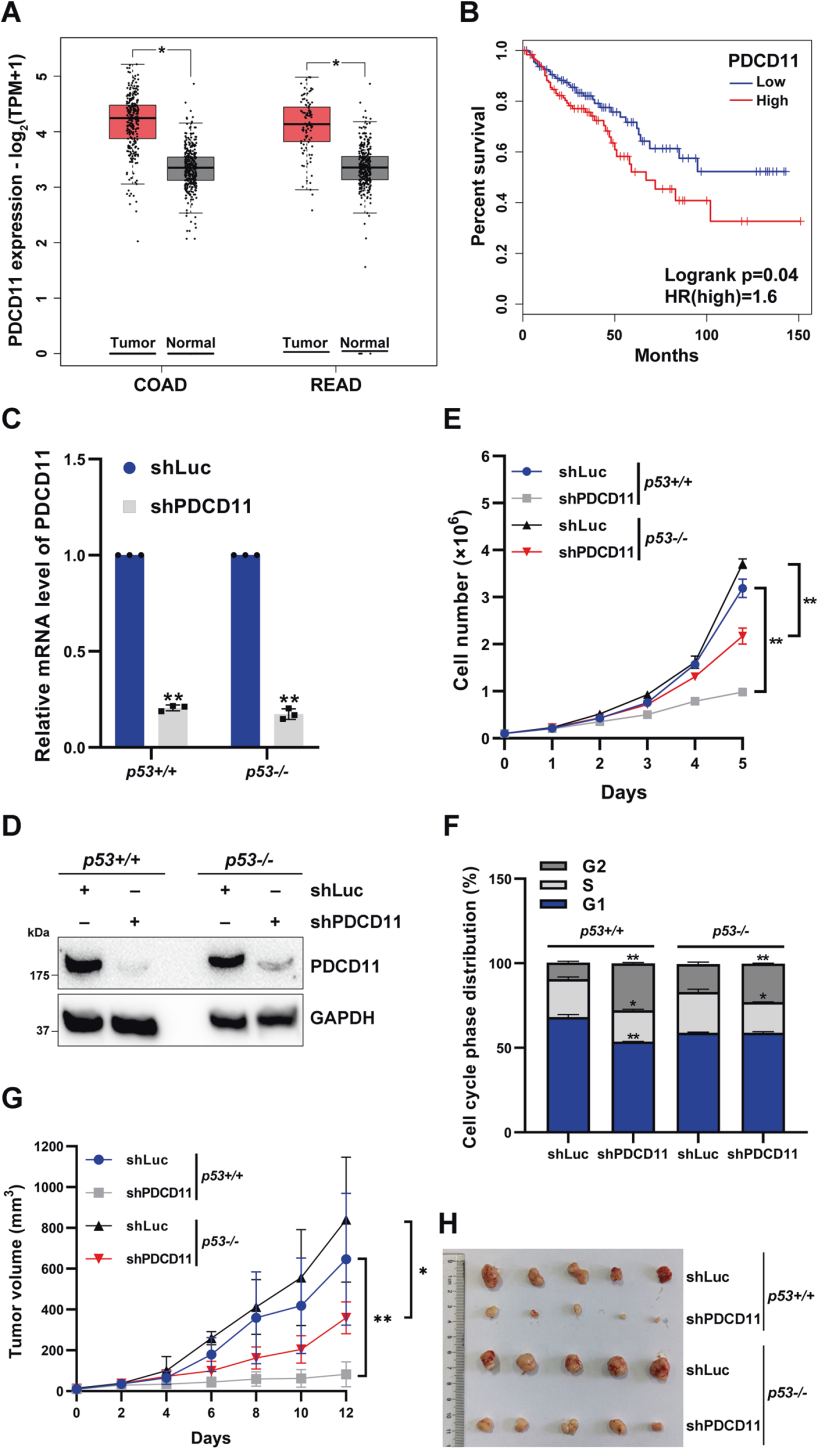
High level of PDCD11 accelerates colorectal cancer progression. **A** GEPIA2 was used to compare the expression level of PDCD11 in colorectal tumor (COAD + READ) and normal samples. The raw data were obtained from TCGA and the GTEx databases. FC ≥ 1.5 and q-value < 0.001 denote significant difference (*). **B** High-level PDCD11 indicates a high risk (HR = 1.6) of shortening the overall survival time of CRC patients (Logrank *p* = 0.04). Data were obtained from TCGA database and analyzed using GEPIA2. **C**–**F** All the lentivirus-transduced HCT116 cells were treated with Doxy to induce the expression of shRNAs. **C** The mRNA level of PDCD11 in the cells was determined by qRT-PCR (*n* = 3). GAPDH was used as an internal control to normalize the values. The normalized values of the cells expressing shLuc were set to 1. **D** Cellular level of PDCD11 protein was determined by western blotting. GAPDH was used as a loading control. **E** Growth curves of the cells with or without downregulation of PDCD11 (*n* = 3). **F** Cell cycle was determined by PI staining assay (*n* = 3). **G** and **H** Nude mice bearing lentivirus-transduced HCT116 tumors were administered with Doxy in drinking water for 12 days to downregulate the intratumor level of PDCD11. **G** Tumor growth curves in different groups (*n* = 5). **H** Tumor images in different groups when the 12-day course ended. **C** and **E**–**G** Data are shown as mean ± SD. *P < 0.05; **P < 0.01 denote significant difference.

To clarify the role of PDCD11 in cancers, we first compared the expression levels of PDCD11 in cancer and normal tissues by using Gene Expression Profiling Interactive Analysis 2 (GEPIA2) (http://gepia2.cancer-pku.cn/) [22] to analyze the data from TCGA [10] and the GTEx [11] databases. PDCD11 displays a higher level in tumor than in normal samples for several cancer types including CRC (COAD: colon cancer + READ: rectal cancer) (Figs. 1A and S1). Unfortunately, high-level PDCD11 significantly shortened the overall survival time of CRC patients (Logrank *p* < 0.05) (Fig. 1B).

Since positive PDCD11 expression is closely associated with adverse outcome in CRC cases, we constructed stable HCT116 CRC cell strains inducibly expressing shLuc or shPDCD11. Doxy treatment induced an efficient downregulation in mRNA (~80%) (*P* < 0.01) and protein levels of PDCD11 (Fig. 1C, D), thereby declining the cell proliferation rate (Fig. 1E), no matter whether p53 was present or absent. On day 5, the number of PDCD11-downregulated HCT116^*p53−/−*^ cells was decreased to ~60% in comparison with that of the controls (*P* < 0.01). The presence of p53 further decreased the number of PDCD11-downregulated HCT116^*p53+/+*^ cells to ~30%, indicating that PDCD11 promotes cell proliferation in both p53-dependent and -independent manners.

To investigate whether PDCD11 regulates cell proliferation by affecting cell cycle progression, we performed PI staining assay. In HCT116^*p53+/+*^ cells, downregulation of PDCD11 resulted in an increased percentage of G2 population (*P* < 0.01) but a decreased percentage of G1 and S populations (*P* < 0.05) (Fig. 1F), indicating a typical G2/M arrest. Similarly, downregulation of PDCD11 also arrested HCT116^*p53−/−*^ cells in the G2 phase (Fig. 1F). The percentage of G2 population of PDCD11-downregulated *p53*^+*/*+^ cells increased by 186 ± 19%, much higher than the increase rate (38 ± 8%) when p53 was absent, indicating that PDCD11 regulates cell cycle in both p53-dependent and -independent manners.

To further verify the role of PDCD11 in vivo, the cell strains inducibly expressing shRNAs were inoculated into nude mice. Consistent with the in vitro data (Fig. 1E), HCT116 tumors expressing shPDCD11 grew much slower than the controls during a treatment of Doxy (Fig. 1G, H), regardless of the presence or absence of p53. On day 12, downregulation of PDCD11 in *p53*^+*/*+^ tumors led to a growth inhibition rate of ~90%, which was reduced to approximately 60% when p53 was absent, indicating that PDCD11 promoted tumor growth in both p53-dependent and -independent manners.

Collectively, high-level PDCD11 indicates a poor prognosis in CRC and acts as an oncoprotein to accelerate cancer cell growth through facilitating cell-cycle transition from G2 to M phase. The pro-oncogenic function of PDCD11 is related but not entirely attributed to p53.

### PDCD11 promotes cancer cell growth by increasing CDK1 level in a p53-dependent manner and upregulating CDC25C without dependence on p53

**Fig. 2:**
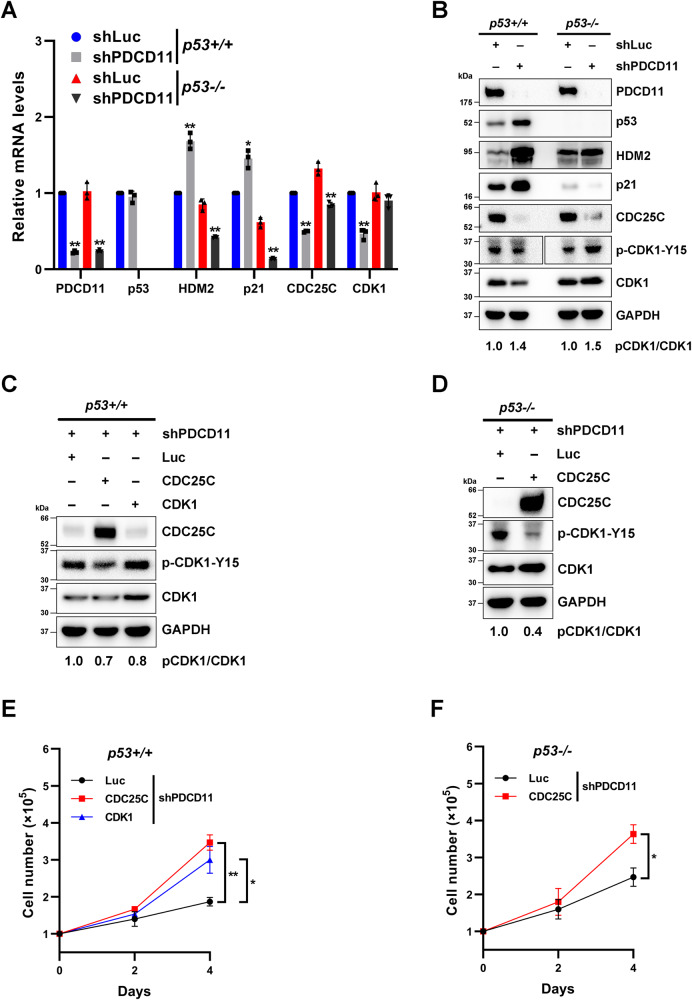
PDCD11 accelerates colorectal cancer cell growth by modulation of p53-CDK1 and CDC25C-CDK1 axes. All the lentivirus-transduced cells were treated with Doxy to induce the expression of shRNAs. **A** The mRNA levels of PDCD11, p53, HDM2, p21, CDC25C, and CDK1 in HCT116 cells were determined by qRT-PCR (*n* = 3). GAPDH was used as an internal control to normalize the values. The normalized values of HCT116^*p53+/+*^ cells expressing shLuc were set to 1. **B** The levels of PDCD11, p53, HDM2, p21, CDC25C, p-CDK1-Y15, and CDK1 proteins in the cells were determined by western blotting. GAPDH was used as a loading control. p-CDK1/CDK1 ratio indicates relative phosphorylation rate of CDK1 and the values of the cells expressing shLuc were set to 1 for normalization. **C**, **D** Western blot analyses were performed to investigate the levels of CDC25C, CDK1, and p-CDK1-Y15 proteins in PDCD11-downreguatled HCT116 cells transfected with pcDNA3-Luc, pcDNA3-CDC25C, or pcDNA3-CDK1. GAPDH was used as a loading control. p-CDK1/CDK1 ratio indicates relative phosphorylation rate of CDK1 and the values of the cells expressing Luc were set to 1 for normalization. **E**, **F** Cell growth curves of PDCD11-downregulated HCT116 cells transfected with pcDNA3 plasmids (*n* = 3). **A**, **E**, and **F** Data are shown as mean ± SD. *P < 0.05; **P < 0.01 denote significant difference.

Since our data show that p53 is connected to the tumor-promoting effect of PDCD11 (Fig. 1), we wondered how p53 is involved. To address this question, we analyzed the mRNA and protein levels of p53 and its canonical target genes p21 and HDM2 [23]. Downregulation of PDCD11 did not affect the mRNA level of p53 in HCT116^*p53+/+*^ cells, but increased its protein level, indicating that PDCD11 downregulates p53 protein but not p53 transcripts (Fig. 2A, B). Hence, the mRNA and protein levels of p21 and HDM2 were elevated in PDCD11-downregulated *p53*+*/*+ cells (Fig. 2A, B), indicating that PDCD11 inhibits the expression of p21 and HDM2 at both mRNA and protein levels in the presence of p53. However, downregulation of PDCD11 led to a decreased mRNA level of p21 and HDM2 in *p53-/-* cells (Fig. 2A), indicating that PDCD11 might be shifted to maintain p21 and HDM2 mRNA levels when p53 was absent.

To investigate how downregulation of PDCD11 resulted in G2/M arrest (Fig. 1F), we analyzed the mRNA and protein levels of CDC25C and CDK1, which commonly participate in the regulation of G2/M checkpoint [24, 25]. In either HCT116^*p53+/+*^ or HCT116^*p53-/-*^ cells, reduction of PDCD11 expression decreased the mRNA (*P* < 0.01) and protein levels of CDC25C (Fig. 2A, B), indicating that PDCD11 upregulates CDC25C in a p53-independent manner. Downregulation of PDCD11 also reduced the mRNA (*P* < 0.01) and protein levels of CDK1 in *p53*+*/*+ cells but not when p53 was absent (Fig. 2A, B). The data indicate that PDCD11 upregulates CDK1 by downregulation of p53, which was consistent with the publication in which p53 inhibits the transcription of CDK1 [26]. Since CDC25C is commonly overexpressed in cancer cells to facilitate G2/M transition by dephosphorylating CDK1 [24, 25], we assessed the phosphorylation level of CDK1 at Y15 (p-CDK1-Y15). Unsurprisingly, the decreased CDC25C level in either *p53*+*/*+ or *p53−/−* cells led to an increased ratio of p-CDK1-Y15/CDK1 (Fig. 2B). To further confirm the dephosphorylation of CDK1 by CDC25C, pcDNA3-CDC25C plasmid were transfected into PDCD11-downregulated cells. Overexpression of CDC25C decreased the ratio of pCDK1/CDK1 in *p53*+/+ and *p53−/−* cells with downregulation of PDCD11 (Fig. 2C, D), leading to an accelerated proliferation (Fig. 2E, F). These findings indicate that PDCD11 positively regulates the CDC25C-CDK1 axis to promote G2/M transition and proliferation of CRC cells in a p53-independent manner. Moreover, overexpression of CDK1 also increased the proliferation rate of HCT116^*p53+/+*^ cells with downregulated PDCD11 (Fig. 2C, E), showing that PDCD11 removes p53-mediated transcriptional inhibition of CDK1, thereby coordinating with CDC25C to accelerate G2/M transition and proliferation.

To explore why PDCD11 and RSL1D1 regulate different cell cycle checkpoints, we assessed the level of CDK1 protein in RSL1D1-downregulated cells. Different from the outcome of PDCD11 silencing, the CDK1 level was unchanged in response to the upregulated p53 by RSL1D1 silencing (Fig. S2). This can help explain why RSL1D1 negatively regulates p53 to facilitate G1/S transition but not G2/M [6].

Collectively, PDCD11 promotes CRC cell proliferation by increase of CDK1 expression in a p53-dependent manner and upregulation of CDC25C in a p53-independent manner. Through modulation of p53-CDK1 and CDC25C-CDK1 axes, PDCD11 helps CRC cells pass G2/M checkpoint.

### PDCD11 promotes cancer cell survival by resisting DNA damage stress-induced G2/M arrest and apoptosis

**Fig. 3:**
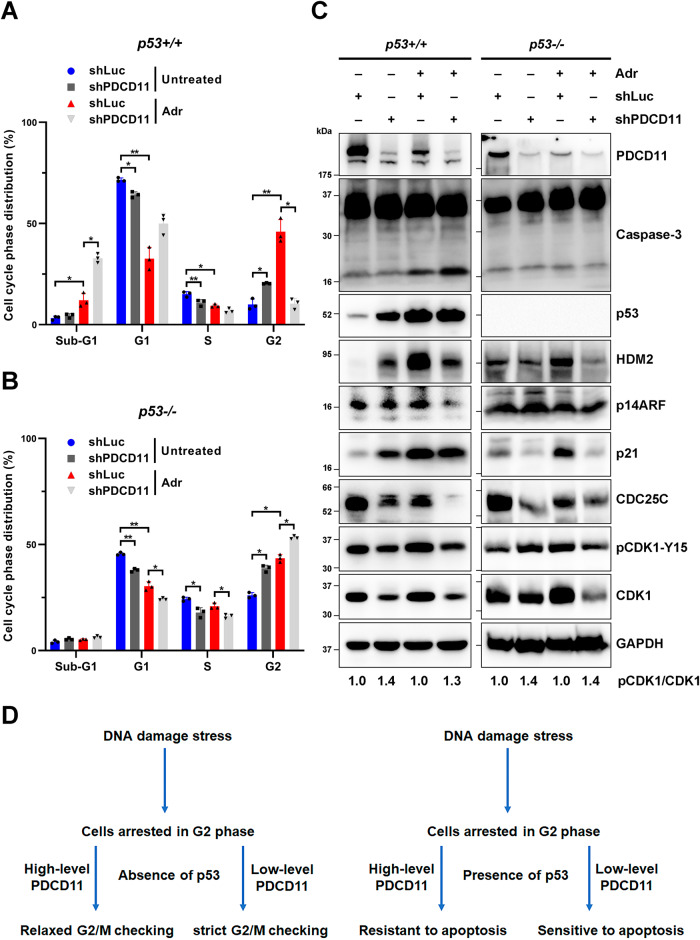
Downregulation of PDCD11 sensitizes cells to DNA damage stress-induced G2/M arrest and apoptosis. All the lentivirus-transduced HCT116 cells were treated with Doxy to induce expression of shRNAs. Prior to be harvested, the cells were untreated or treated with 1 μM Adr for 24 h. **A**, **B** Cell cycle was determined by PI staining assay (*n* = 3). Sub-G1 indicates the apoptotic cell population. **C** The levels of PDCD11, Caspase-3 (full-length and cleaved p17), p53, HDM2, p14ARF, p21, CDC25C, p-CDK1-Y15, and CDK1 proteins in the cells were determined by western blotting. GAPDH was used as a loading control. p-CDK1/CDK1 ratio indicates relative phosphorylation rate of CDK1 and the values of the cells expressing shLuc were set to 1 for normalization. **D** A model showing the role of PDCD11 in response to DNA damage stress (DDS). In the absence of p53, PDCD11 relaxes the G2/M checkpoint when cells were arrested in G2 phase by DDS. In the presence of p53, PDCD11 turns to resist DDS-induced apoptosis of G2-phase cells.

We also explored the role of PDCD11 when cells were already arrested in G2 phase by adriamycin (Adr)-induced DDS [27]. In *p53−/−* cells, percentage of G2 population was increased by Adr treatment (*P* < 0.05) and further increased by downregulation of PDCD11 (*P* < 0.05), indicating an enhanced G2/M arrest (Fig. 3B, D). Neither the sub-G1 (apoptotic) population nor p17 level (an apoptotic marker cleaved from full-length Caspase-3) [28] was affected when p53 was absent (Fig. 3B, C). In contrast, Adr treatment to *p53*+*/*+ cells remarkably upregulated the two apoptotic markers, more than inducing G2/M arrest (Fig. 3A, C). Downregulation of PDCD11 further increased the sub-G1 (*P* < 0.05) and p17 levels but decreased the proportion of cells in G2 phase (*P* < 0.05) (Fig. 3A, C). Obviously, the gain of sub-G1 population resulted from the loss of G2 population (Fig. 3A), revealing that reduction of PDCD11 level sensitized G2-phase cells to DDS-induced apoptosis in a p53-dependent manner (Fig. 3D).

To explore how downregulation of PDCD11 enhanced Adr-induced G2/M arrest in *p53−/−* cells, we assessed the levels of CDC25C, CDK1, and pCDK1 proteins upon Adr treatment. Consistent with the data in untreated cells, PDCD11 still can upregulate CDC25C to dephosphorylate CDK1 and thereby accelerate G2/M transition under DDS (Fig. 3B, C). Interestingly, DDS endowed PDCD11 to upregulate CDK1 when p53 was absent, also a contribution to G2/M transition (Fig. 3B, C). Since downregulation of PDCD11 sensitized cells to Adr-induced apoptosis in *p53*+*/*+ cells (Fig. 3A, C), we assessed the protein levels of p53 and its target genes upon Adr treatment. Unlike in untreated cells, downregulation of PDCD11 did not affect the p53 level but decreased the levels of HDM2 and p21 in a p53-independent manner (Fig. 3C). These findings suggest that PDCD11 resists DDS-induced apoptosis in a p53-dependent manner but not via regulating the level of p53 protein or its transcription factor activity.

Collectively, PDCD11 exhibited a pro-survival function under DDS. In the absence of p53, PDCD11 upregulates CDC25C and CDK1 to relax the G2/M checkpoint in response to DDS-induced G2/M arrest. Upon the presence of p53, PDCD11 is shifted to help G2-phase cells resist DDS-induced apoptosis.

### PDCD11 ubiquitinates and degrades p53 in a HDM2-dependent manner

**Fig. 4:**
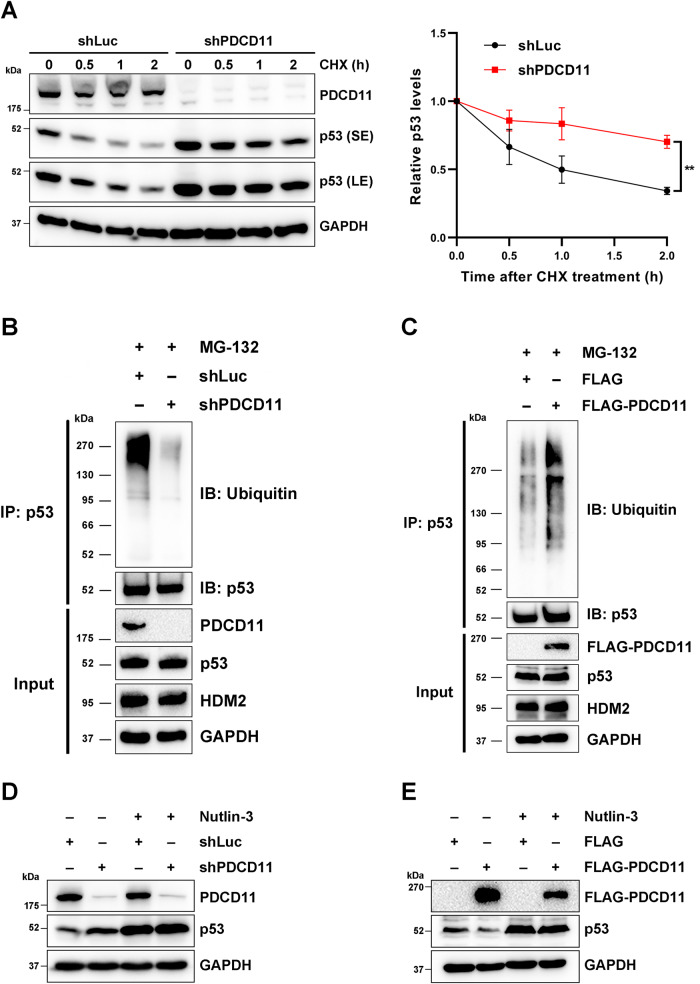
PDCD11 relies on HDM2 to ubiquitinate and degrade p53. **A**, **B** and **D** All the lentivirus-transduced cells were treated with Doxy to induce expression of shRNAs. **C**, **E** Cells were transfected with pBiFC-mCherryN159-FLAG or pBiFC-mCherryN159-FLAG-PDCD11. **A** Western blot analyses were performed to determine the levels of PDCD11 and p53 proteins in HCT116^*p53+/+*^ cells treated with 100 µg/mL CHX for indicated times. To facilitate comparison, p53 bands were obtained through a short exposure (SE) for shPDCD11 or long exposure (LE) for shLuc. GAPDH was used as a loading control. Relative quantification of the p53 levels was shown (*n* = 3). The values of the cells at 0 h were set to 1. **B**, **C** Ubiquitinated and non-ubiquitinated p53 was immunoprecipitated using anti-p53 (DO-1) from HCT116^*p53+/+*^ cells treated with MG-132 (20 µM, 8 h). Anti-Ubiquitin (P4D1) and anti-p53 (DO-1) were used to detect ubiquitinated and non-ubiquitinated p53, respectively. The levels of PDCD11/FLAG-PDCD11, p53, and HDM2 proteins in the input cell lysate were determined by western blotting and GAPDH was used as a loading control. **D**, **E** Western blot analyses were performed to determine the levels of PDCD11/FLAG-PDCD11 and p53 proteins in HCT116^*p53+/+*^ cells untreated or treated with Nutlin-3 (40 µM, 12 h). GAPDH was used as a loading control.

Since the level of wild-type p53 is kept low due to its instability in CRC cells [29], we performed CHX chase assay [30] to compare p53 protein stability in PDCD11-downregulated and control cells. Downregulation of PDCD11 significantly delayed p53 degradation (*P* < 0.01), indicating that PDCD11 destabilizes p53 protein (Fig. 4A).

Considering that ubiquitin-proteasome pathway is the main route to degrade p53 in the cells [31], we assessed the ubiquitination level of p53 in HCT116^*p53+/+*^ cells treated with proteasome inhibitor MG-132 [24]. Downregulation of PDCD11 remarkably decreased the level of ubiquitinated p53 (Fig. 4B), whereas overexpression of PDCD11 increased the level of ubiquitinated p53 (Fig. 4C), indicating that PDCD11 degrades p53 by ubiquitination. Since p53 ubiquitination is majorly regulated by HDM2 [31], we treated the cells with Nutlin-3, a highly selective HDM2 inhibitor [32]. Unlike in untreated cells, the level of p53 protein no longer showed an increase in PDCD11-downregulated cells when p53-HDM2 interaction was blocked by Nutlin-3 (Fig. 4D). Similarly, overexpressed PDCD11 led to a decreased p53 level in untreated cells, but rarely affecting the level of p53 in the cells treated with Nutlin-3 (Fig. 4E). These findings indicate that PDCD11 downregulates p53 in a HDM2-dependent manner. By the way, p53 levels in the cells were increased by downregulation of PDCD11 and further increased by Nutlin treatment, indicating that PDCD11 deficiency did not completely deprive the HDM2 function (Fig. 4D) because some other factors also help HDM2 to degrade p53 [6, 33, 34].

Collectively, PDCD11 negatively regulates p53 through HDM2-mediated ubiquitination and proteasomal degradation.

### PDCD11 interacts with p53 and HDM2 in the nucleoplasm

**Fig. 5:**
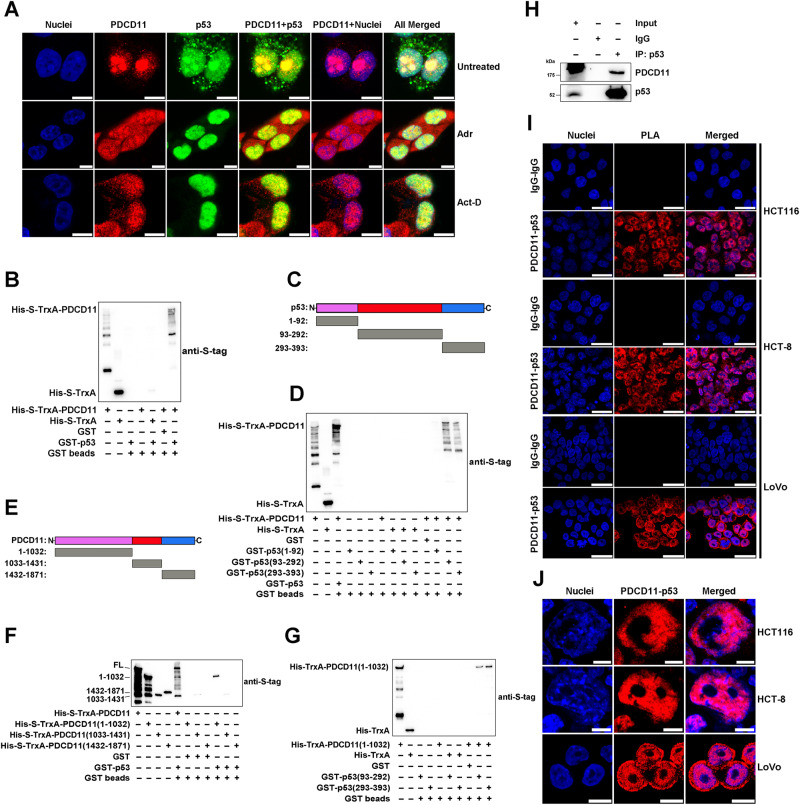
PDCD11 interacts with p53 in the nucleoplasm. **A** IF assay was performed to investigate the subcellular localization of endogenous PDCD11 (red), p53 (green), and their colocalization (yellow) in HCT116^*p53+/+*^ cells untreated or treated with Adr (1 µM, 24 h) or Act-D (5 nM, 24 h). The nuclei were stained to blue. Scale bars: 10 µm. **B**–**G** GST pulldown assay was performed to investigate PDCD11-p53 interaction in vitro and identify the binding domains. Full-length PDCD11 (PDCD11-FL) and its truncated variants were His-S-tagged, whereas p53-FL and its truncations were GST-tagged. **B** PDCD11-FL interacts with p53-FL in vitro. **C** Schematic diagram showing the construction of truncations of p53. **D** PDCD11-FL interacts with p53 (93-292) and p53 (293-393). **E** Schematic diagram showing the construction of truncations of PDCD11. **F** PDCD11 (1-1032) interacts with p53-FL. **G** PDCD11 (1-1032) interacts with p53 (93-292) and p53 (293-393). **H** Co-IP assay using anti-p53 (DO-1) was performed to investigate PDCD11-p53 interaction in HCT116^*p53+/+*^ cells. Normal mouse IgG was used as a negative control. Input represents 5% of the cell lysate utilized for IP. PLA was performed to investigate the formation (**I**) and localization (**J**) of endogenous PDCD11-p53 complex (red) in a panel of CRC cells harboring wild-type p53. The nuclei were stained to blue. Scale bars: 25 µm (**I**) or 10 µm (**J**).

**Fig. 6:**
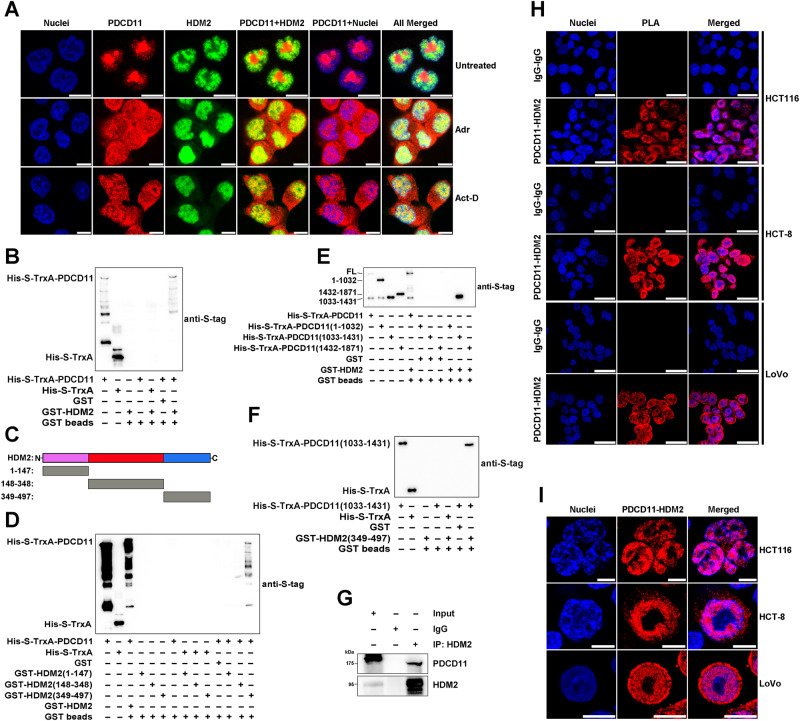
PDCD11 interacts with HDM2 in the nucleoplasm. **A** IF assay was performed to investigate the subcellular localization of endogenous PDCD11 (red), HDM2 (green), and their colocalization (yellow) in HCT116^*p53+/+*^ cells untreated or treated with Adr (1 µM, 24 h) or Act-D (5 nM, 24 h). The nuclei were stained to blue. Scale bars: 10 µm. **B**–**F** GST pulldown assay was performed to investigate PDCD11-HDM2 interaction in vitro and identify the binding domains. PDCD11-FL and its truncations were His-S-tagged, whereas HDM2-FL and its truncations were GST-tagged. **B** PDCD11-FL interacts with HDM2-FL in vitro. **C** Schematic diagram showing the construction of truncations of HDM2. **D** PDCD11-FL interacts with HDM2 (349-497). **E** PDCD11 (1033-1431) interacts with HDM2-FL. **F** PDCD11 (1033-1431) interacts with HDM2 (349-497). **G** Co-IP assay using anti-HDM2 (D1V2Z) was performed to investigate PDCD11-HDM2 interaction in HCT116^*p53+/+*^ cells. Normal rabbit IgG was used as a negative control. Input represents 5% of the cell lysate utilized for IP. PLA was performed to investigate the formation (**H**) and localization (**I**) of endogenous PDCD11-HDM2 interaction (red) in a panel of CRC cells harboring wild-type p53. The nuclei were stained to blue. Scale bars: 25 µm (**H**) or 10 µm (**I**).

In either untreated or MG-132-treated HCT116^*p53+/+*^ cells, the level of HDM2 protein did not show a reduction in response to downregulation of PDCD11 (Figs. 2B, 4B), indicating that PDCD11 does not ubiquitinate p53 by regulation of HDM2 level. More likely, PDCD11 regulates p53 through interaction with p53 and/or HDM2. To verify this, we first performed IF assay using a validated homemade anti-PDCD11 (Fig. S3) to investigate the subcellular localization of PDCD11. In a panel of CRC cells harboring wild-type p53 [35, 36], PDCD11 was mainly localized in the nucleolus but also distributed in the nucleoplasm and cytoplasm (Fig. S4). Since stress conditions commonly induce a translocation of nucleolar proteins to the nucleoplasm [1–5], we also investigated the subcellular localization of PDCD11 under Adr-induced DDS or Act-D-induced nucleolar stress. Treatment of either Adr or Act-D induced nucleolar export of PDCD11, resulting in a homogeneous distribution of PDCD11 throughout the whole cell (Fig. 5A). PDCD11 no longer regulated p53 under DDS (Fig. 3C) but still can negatively regulate p53 under nucleolar stress (Fig. S5), indicating that this regulation was independent of PDCD11 translocation. Interestingly, the level of p14ARF, a nucleolar protein to stabilize p53 by inhibiting HDM2 [37], was decreased by nucleolar stress (Fig. S5). This might be a self-protective mechanism in response to the high-level p53 upon stress conditions. Upon DDS, the p14ARF level was decreased in response to PDCD11 silencing in *p53*+*/*+ but not *p53−/−* cells, which might neutralize the p53-stabilizing effect by downregulation of PDCD11, leading to an unchanged p53 level (Fig. 3C). Under either non-stress or stress condition, PDCD11 was colocalized with p53 and HDM2 in both the nucleolus and nucleoplasm (Figs. 5A and 6A). These data suggest that PDCD11-p53 and PDCD11-HDM2 complexes might exist in CRC cells regardless of exogenous stresses.

To verify whether PDCD11 interacts with p53, His-S-tagged PDCD11 and GST-tagged p53 was expressed and purified (Fig. S6A, B) for GST pulldown assay. The results substantiate that PDCD11 interacted with p53 in vitro (Fig. 5B). To identify the PDCD11-binding domain on p53 and the p53-binding domain on PDCD11, we constructed a panel of truncated variants of PDCD11 and p53 proteins (Figs. 5C, E, S6A, B). GST pulldown analysis showed that aa 93-292 and aa 293-393 of p53 interacted with PDCD11, whereas aa 1-1032 of PDCD11 interacted with p53, indicating that PDCD11-p53 complex was formed through the binding between PDCD11 (1-1032) and p53 (93-393) (Fig. 5C–G). To verify PDCD11-p53 interaction in vivo, we performed BiFC assay by co-transfection with pBiFC-mCherryN159-PDCD11 and pBiFC-mCherryC160-p53 plasmids. The results confirm the interaction between overexpressed PDCD11 and p53 in HCT116 cells (Fig. S7A). We further confirmed the endogenous PDCD11-p53 interaction in the cells by performing co-IP (Fig. 5H) and PLA (Fig. 5I, J). The PLA images indicate that naturally formed PDCD11-p53 complex was mainly localized in the nucleoplasm but not the nucleolus (Fig. 5I, J).

To test whether PDCD11 interacts with HDM2, we also performed GST pulldown, BiFC, co-IP, and PLA. GST-pulldown results show that PDCD11 interacted with HDM2 in vitro (Figs. 6B, S6A, C). aa 349-497 of HDM2 was identified to be PDCD11-binding domain, whereas aa 1033-1431 of PDCD11 was identified to be HDM2-binding domain (Figs. 6C-F, S6A, C). BiFC, co-IP, and PLA further confirmed PDCD11-HDM2 interaction in vivo (Figs. 6G–I and S7B). Similar to PDCD11–p53 complex, PDCD11-HDM2 complex was also mainly distributed in the nucleoplasm but not the nucleolus (Fig. 6H and I), although PDCD11 was colocalized with p53 and HDM2 in both the nucleoplasm and nucleolus (Figs. 5A and 6A). The nucleoplasm-specific PDCD11-p53/HDM2 interaction is probably attributed to the different functions of PDCD11 in the nucleoplasm and nucleolus. PDCD11 reportedly regulates mature of 18S rRNA through protein-RNA interaction in the nucleolus [17]. Since most of the RNA binding sites were localized on the p53 binding domain (aa 1-1032) and HDM2-binding domain (aa 1033-1431) of PDCD11 [16], a mass of RNA molecules might competitively inhibit PDCD11-p53/HDM2 interaction in the nucleolus. When in the nucleoplasm, PDCD11 is released from RNA binding and thereby performs its non-nucleolar function through binding to p53, HDM2, or other molecules.

Collectively, PDCD11 directly interacts with both p53 and HDM2 mainly in the nucleoplasm of CRC cells.

### PDCD11 recruits p53 to HDM2 for ubiquitination-mediated p53 degradation

**Fig. 7:**
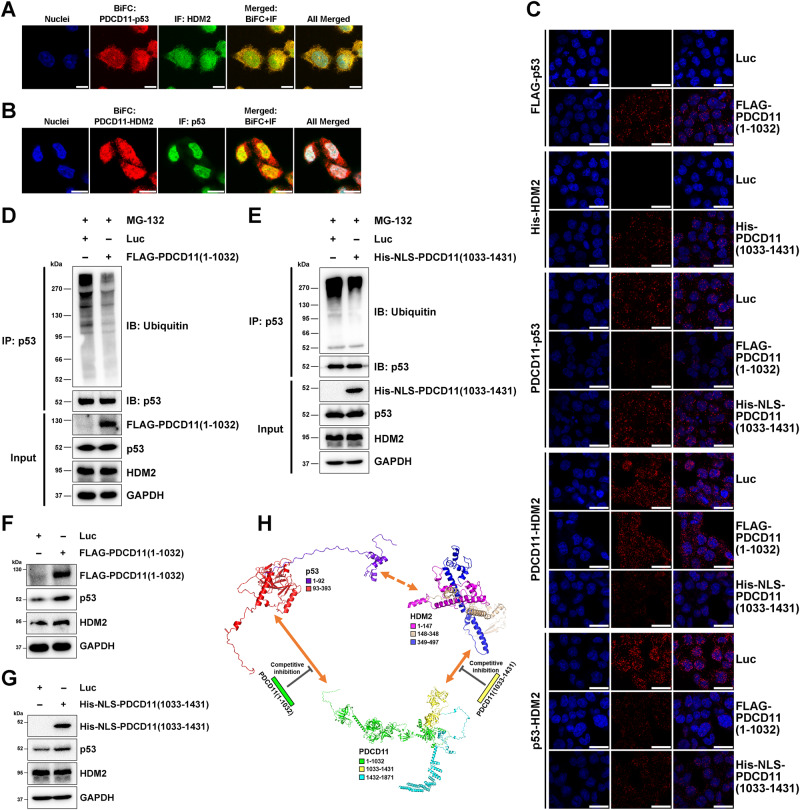
PDCD11 negatively regulates p53 by recruiting p53 to HDM2. Combinational BiFC-IF assay was performed to investigate the intracellular colocalization of PDCD11, p53, and HDM2. HCT116^*p53-/-*^ cells co-transfected with pBiFC-mCherryN159-PDCD11 and pBiFC-mCherryC160-p53 were subjected to IF assay using anti-HDM2 (**A**), whereas HCT116^*p53+/+*^ cells co-transfected with pBiFC-mCherryN159-PDCD11 and pBiFC-mCherryC160-HDM2 were immunostained by anti-p53 (**B**). Red fluorescence indicates a PDCD11-p53/PDCD11-HDM2 complex and green fluorescence indicates endogenous HDM2/p53. Yellow color indicates the colocalization of the three proteins. The nuclei were stained to blue. Scale bars: 10 µm. **C**–**H** All the lentivirus-transduced HCT116^*p53+/+*^ cells were treated with Doxy to induce expression of Luc, FLAG-PDCD11 (1-1032), or His-NLS-PDCD11 (1033-1431) in HCT116^*p53+/+*^ cells. **C** PLA was performed to investigate PDCD11 (1-1032)-p53, PDCD11 (1033-1431)-HDM2, PDCD11-p53/HDM2, and p53-HDM2 interactions (red) in the cells treated with MG-132 (20 µM, 8 h). The nuclei were stained to blue. Scale bars: 25 µm. **D**, **E** Ubiquitinated and non-ubiquitinated p53 was immunoprecipitated using anti-p53 (DO-1) from the cells treated with MG-132 (20 µM, 8 h). Anti-ubiquitin (P4D1) and anti-p53 (DO-1) were used to detect ubiquitinated and non-ubiquitinated p53, respectively. The levels of FLAG-PDCD11 (1-1032)/His-NLS-PDCD11 (1033-1431), p53, and HDM2 proteins in the input cell lysate were determined by western blotting and GAPDH was used as a loading control. **F**, **G** Western blot analyses were performed to determine the levels of FLAG-PDCD11 (1-1032)/His-NLS-PDCD11 (1033-1431), p53, and HDM2 proteins in the cells expressing Luc, FLAG-PDCD11 (1-1032), or His-NLS-PDCD11 (1033-1431). GAPDH was used as a loading control. **H** A model showing the formation of PDCD11-p53-HDM2 complex, which can be competitively destroyed by overexpression of either PDCD11 (1-1032) or PDCD11 (1033-1431).

The N-terminal domain (aa 15-29) of p53 reportedly interacts with the N-terminus of HDM2 (1-125) [38]. PDCD11 (1-1032) and PDCD11 (1033-1431) interacted with p53 (93-393) and HDM2 (349-497), respectively (Figs. 5 and 6). Considering the colocalization of the three proteins with respective binding domains non-overlapped, we speculated that PDCD11 might recruit p53 to HDM2 (Fig. 7H), thereby ubiquitinating and degrading p53. To verify this, we first performed a combinational BiFC-IF assay and found that PDCD11-p53 complex was colocalized with HDM2 in HCT116 cells (Fig. 7A). In addition, we also found that PDCD11-HDM2 complex was colocalized with p53 in the cells (Fig. 7B). These findings indicate that PDCD11, p53, and HDM2 might form a transient triple protein complex.

To further confirm our speculation, we performed competitive inhibition assays. An online tool (https://www.novopro.cn/tools/nls-signal-prediction.html) [39] was used to predict whether the p53-binding domain (aa 1-1032) or HDM2-binding domain (aa 1033-1431) of PDCD11 possesses a nuclear localization signal (NLS) sequence, thereby facilitating us to adopt optimized competitive inhibitors. The results show a NLS sequence (aa 43-69) on PDCD11 (1-1032) but none on PDCD11 (1033-1431). Since the PDCD11-p53 and PDCD11-HDM2 complexes were both formed in the nucleus (Figs. 5J and 6I), we overexpressed PDCD11 (1-1032) with an endogenous NLS sequence and PDCD11 (1033-1431) fused with a SV40-derived NLS [40] to affect PDCD11-p53 and PDCD11-HDM2 interaction, respectively. Interestingly, PDCD11 (1-1032) was not confined to the nucleus but also in the cytoplasm of HCT116 cells (Fig. S8A), whereas NLS-PDCD11 (1033-1431) was distributed in the nucleoplasm and cytoplasm but rarely in the nucleolus (Fig. S8B). We also identified that PDCD11 (aa 1432-1871) contains the epitope of our homemade monoclonal anti-PDCD11 (Fig. S9), ensuring that this antibody specially recognizes endogenous PDCD11 but not the truncated domains (aa 1-1032 or 1033-1431). Unsurprisingly, the overexpressed PDCD11 (1-1032) or PDCD11 (1033-1431) interacted with p53 or HDM2, respectively, to competitively inhibit p53/HDM2 from binding to PDCD11, leading to an attenuated p53-HDM2 interaction (Fig. 7C). This therefore reduced HDM2-mediated p53 ubiquitination (Fig. 7D, E) to upregulate p53 (Fig. 7F, G), indicating that the enhanced p53-HDM2 interaction by PDCD11 was destroyed by introducing the truncated p53- or HDM2-binding domain of PDCD11 (Fig. 7H).

Collectively, PDCD11 recruits p53 to HDM2 through direct interaction, thereby forming a triple protein complex to enhance HDM2-mediated ubiquitination and degradation of p53.

## Discussion

**Fig. 8:**
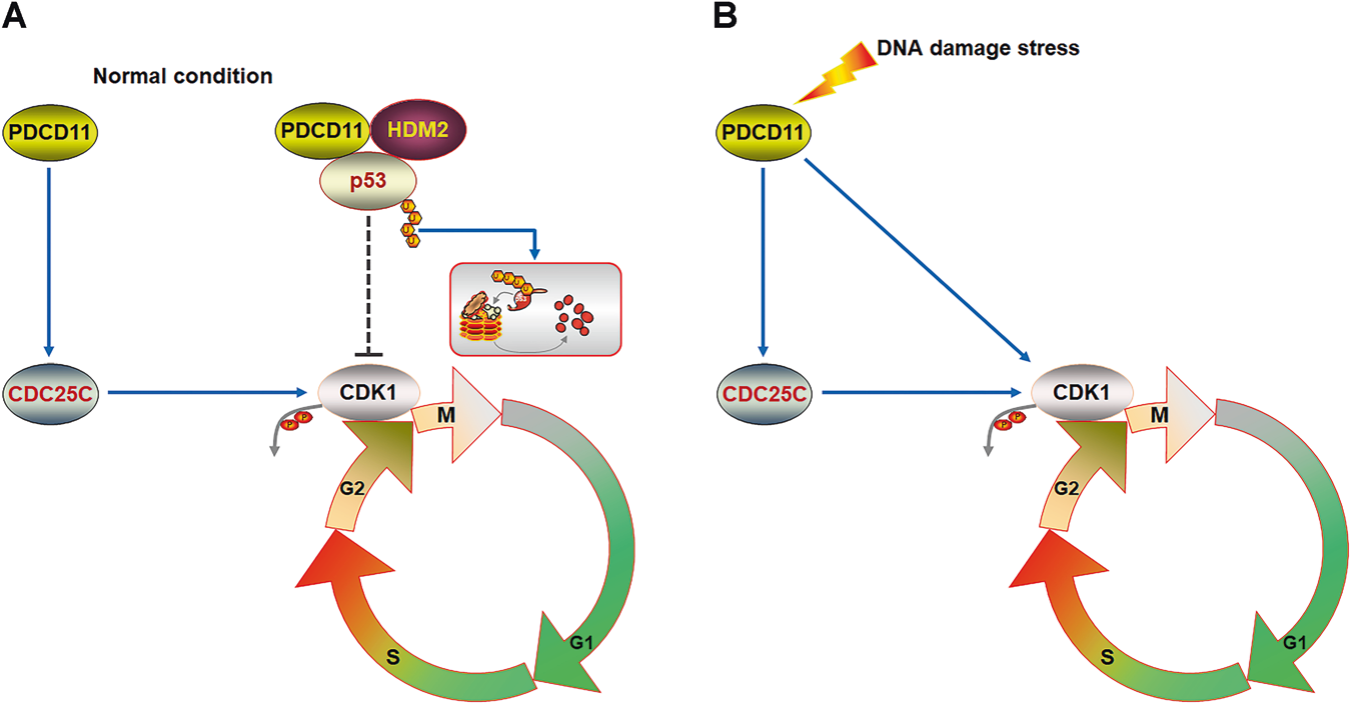
A proposed regulatory model showing how PDCD11 facilitates G2/M transition in colorectal cancer cells. **A** On one hand, PDCD11 recruits p53 to HDM2 for ubiquitination, thereby degrading p53 to upregulate CDK1 to help CRC cells pass G2/M checkpoints under normal conditions. On the other hand, PDCD11 upregulates CDC25C to dephosphorylate CDK1, leading to an accelerated G2/M transition and proliferation in a p53-independent manner. **B** In addition to upregulating CDC25C to dephosphorylate CDK1, PDCD11 turns to upregulate CDK1 independently of p53 to resist DDS-induced G2/M arrest.

p53, the most studied tumor suppressor, is mutated in ~50% of cancer cases but presents as wild type in the other half [41]. HDM2 acts as the main factor to maintain wild-type p53 at a low level, thereby facilitating growth and survival of cancer cells without p53 mutation [33]. Several other factors, such as MDM4 (or MDMX) and UbcH5c, also help cancer cells inhibit p53 directly or by enhancing HDM2 function [33, 34]. Only one of these factors can not completely govern the p53-HDM2 interaction, but joins forces with the others to restrict p53. Recently, we added a new member RSL1D1 to the panel of HDM2 enhancers and p53 inhibitors [6]. This nucleolar protein not only interacts with and stabilizes HDM2 transcripts but also recruits p53 to HDM2 for ubiquitination and degradation in a cell-type-specific manner [6], thereby contributing to the low-level p53 in CRC cells [29]. In the current study, we found another nucleolar protein PDCD11, which also recruits p53 to HDM2 through direct interaction with the two proteins, thereby degrading p53 in CRC cells (Fig. 8A). Other than the regulation of G1/S checkpoint by RSL1D1 in HCT116 cells [6], PDCD11 helps cancer cells pass G2/M checkpoint by regulating the p53-CDK1 and CDC25C-CDK1 axes (Fig. 8A).

Our previous [6] and present studies demonstrate that these two nucleolar proteins, RSL1D1 and PDCD11, with “extra-nucleolar” localization in CRC cells, help HDM2 negatively regulate p53. Most of nucleolar proteins are rarely distributed outside the nucleolus unless facing stress conditions. In response to nucleolar stress, nucleolar proteins, such as RPL5, RPL11, RPL23, NPM1, GLTSCR2, and ARF, were translocated to the nucleoplasm, leading to an inhibitory binding to HDM2 and thus activation of the p53 pathway [42–44]. Unlike most other nucleolar proteins, RSL1D1 [6] and PDCD11 (Figs. 1A, 5, 6, S1, and S4) possess an altered distribution due to their high levels in CRC cells. They were colocalized with p53 and HDM2 in both the nucleolus and nucleoplasm of CRC cells. Another difference of RSL1D1 and PDCD11 from most other nucleolar proteins is their direct binding capacity to p53. RSL1D1 [6] and PDCD11 (Figs. 5–7) not only directly interact with HDM2 but also with p53, which facilitates to recruit p53 to HDM2 to form triple protein complexes. This enhances p53-HDM2 interaction, facilitating to ubiquitinate and degrade p53 and thereby promoting tumor progression (Figs. 1 and 7) [6]. More importantly, these findings suggest that “extra-nucleolar” RSL1D1 and PDCD11 should be potential tumor markers in CRC theranostics.

Although RSL1D1 and PDCD11 similarly regulate p53 through interaction with p53 and HDM2, they probably regulate the p53-HDM2 loop in differential manners. Unlike PDCD11, RSL1D1 can directly increase the HDM2 level to degrade p53 [6]. Since RSL1D1 [5] and PDCD11 (Fig. 6) interacts with the same region (aa 349-497) of HDM2 and the RSL1D1- (aa 93-292)[6] and PDCD11-binding (93-393) (Fig. 5) domains on p53 were overlapped, the four proteins are unlikely to form a tetrad protein complex. More likely, RSL1D1 and PDCD11 recruit p53 to HDM2 without help from each other, thereby affecting cell-cycle machinery in differential manners. RSL1D1 regulates p53-p21 but not p53-CDK1 axis to help HCT116 cells pass G1/S checkpoint (Fig. S2) [6], whereas PDCD11 prefers to promote G2/M transition by regulating the p53-CDK1 and CDC25C-CDK1 axes, although PDCD11 also regulates p53-p21 (Figs. 1 and 2). More than a G1/S inhibitor, p21 sometimes also coordinates with p53 to inhibit CDK1 and thereby contributes to G2/M arrest [45]. This might help explain why the upregulated p53-p21 by PDCD11 silencing did not cause G1/S arrest. Since RSL1D1 and PDCD11 synergistically leading to an abnormal activity of cell-cycle machinery by relaxing G1/S and G2/M checking, deficiency of either of them will arrest cell proliferation. Accordingly, RSL1D1/PDCD11 should be defined as a pair of key driving factors to force CRC tumorigenesis and development.

Recent studies reveal that deregulation or overexpression of cell-cycle proteins, such as CDC25C, Cyclin A/B, Cyclin D/E, Cyclin H, CDK1/2, CDK4/6, CDK7, and CDC7, participate in activation of a wide range of tumorigenic signaling, in addition to regulation of cell division [9]. With a clinical consideration of CDK4/6 inhibitors [9, 46, 47], targeting these cell-cycle proteins is increasingly seen as an effective anticancer strategy. For instance, CDC25C inhibitor M2N12 [48] and CDK1 inhibitor RO-3306 [49, 50] are both given attention due to their clinical potentials. Since the present study demonstrates that PDCD11 regulates G2/M checkpoint through modulation of p53-CDK1 and CDC25C-CDK1 signaling pathways (Figs. 1 and 2), we believe that PDCD11 is a key node in the cell-cycle networks, also a potential target for CRC therapy. Development of antibodies or small molecule compounds antagonizing PDCD11 might be an effective anticancer strategy to prevent PDCD11 from binding to p53, HDM2, or other effector molecules.

Beyond the pro-growth function under normal condition, PDCD11 also exerts a pro-survival function under DDS (Figs. 3 and 8B). In addition to regulating CDC25C-CDK1 axis, PDCD11 is licensed to regulate the CDK1 level independently of p53, thereby helping the DNA-damaged cells pass G2/M checkpoint. When p53 is present to trigger apoptosis of DNA-damaged cells, PDCD11 turns to desensitize G2-phase cells to apoptotic signals, leading to a defective DNA damage response (DDR). Interestingly, PDCD11 regulates neither the level nor transcription factor activity of p53, but still prevents DDS-induced apoptosis in a p53-dependent manner. Since post-translational modifications of p53 or its interactions with other molecules reportedly affect apoptosis independently of p53 transcription factor activity, PDCD11 is likely to prevent apoptosis through affecting these routes [51–53]. This hypothesis should be probed in our future work. Since defective DDR limits the chemotherapy efficacy and targeting DDR pathways is a promising therapeutic strategy in CRC [54, 55], we believe that inhibition of PDCD11 should sensitize CRC cells to DDR signals and thereby benefit the patients from chemotherapy.

In conclusion, PDCD11 acts as an oncoprotein overexpressed in CRC cells and thereby displays an “extra-nucleolar” localization. This facilitates PDCD11 to interact with p53 and HDM2 in the nucleoplasm, thereby recruiting p53 to HDM2 for ubiquitination. The ensuing low-level p53 leads to an enhanced transcription of CDK1, which is dephosphorylated by high-level CDC25C to help cells pass G2/M checkpoint. Upon DDS, PDCD11 also can upregulate CDK1 independently of p53 to further relax G2/M checking. Through p53-dependent and -independent routes, PDCD11 accelerates colorectal cancer progression and desensitizes cancer cells to DNA-damage agents. These findings highlight that PDCD11 is a potential target for tumor therapy and “extra-nucleolar” PDCD11 should be a potential CRC marker.

## Materials and methods

Detailed materials and methods are available in Supplementary Information.

### Supplementary information

Supplementary Information

## Data Availability

All data generated or analyzed during this study are available from the corresponding authors upon reasonable request.
