# Supplementary material for: Programmed cell death 11 modulates but not entirely relies on p53-HDM2 loop to facilitate G2/M transition in colorectal cancer cells
Source: Oncogenesis. 2023 Dec 7;12(1):57. doi: 10.1038/s41389-023-00501-2 (PMC10703783; doi:10.1038/s41389-023-00501-2)
Supplement: Supplementary file 1 — Supplementary Information [file 41389_2023_501_MOESM1_ESM.docx]

**Supplementary Materials**

**Programmed cell death 11 modulates but not entirely relies on p53-HDM2 loop to facilitate G2/M transition in colorectal cancer cells**

**Li Ding*, Yujie Xu, Lin Xu, Chenhong Zhao, Zhiping Zhang, Jie Zhang, Kai Liao, Yuerou Chen, Jingwen Li, Xinyu Mei, Xinyue Zhang***

*Correspondence:

[liding@yzu.edu.cn](mailto:liding@yzu.edu.cn) (L.D.);

[zhanglabsubmission@163.com](mailto:zhanglabsubmission@163.com) or [zhangxinyue@yzu.edu.cn](mailto:zhangxinyue@yzu.edu.cn) (X.Z.)

This file includes:

MATERIALS AND METHODS

Supplemental Table S1

Supplemental Figure S1

Supplemental Figure S2

Supplemental Figure S3

Supplemental Figure S4

Supplemental Figure S5

Supplemental Figure S6

Supplemental Figure S7

Supplemental Figure S8

Supplemental Figure S9

**MATERIALS AND METHODS**

**Cell culture**

Cell culture methods and the source of HCT116*^p53+/+^*, HCT116*^p53-/-^*, HCT-8, and Lenti-X^TM^ 293T cell lines were described in our previous work [1, 2]. LoVo cells were provided by the Cell Bank of the Chinese Academy of Sciences, and cultured in F-12K medium supplemented with 10% fetal bovine serum at 37 ℃ under a 5% CO_2_ atmosphere.

**Mice**

Female 5-week-old BALB/c*-Foxn1^nu^* nude mice weighing 18~22 g were provided by the Experimental Animal Center of Yangzhou University (Yangzhou, Jiangsu, China). All animal experiments were approved by the Institutional Animal Care and Use Committee of Yangzhou University and performed in compliance with the guidelines of Jiangsu Laboratory Animal Welfare and Ethical of Jiangsu Administrative Committee of Laboratory Animals. The mice were randomly grouped for seeding different cancer cell strains.

After selection with puromycin, lentiviral shLuc/shPDCD11-transduced HCT116 cells (2 × 10^6^/mouse) were subcutaneously injected into upper right axilla of mice. When tumors grew to approximately 3 mm in diameter, the mice were fed with 2 mg/mL doxycycline (Doxy) in drinking water containing 5% sucrose to induce downregulation of PDCD11. Tumor volumes were measured every other day. All the mice were sacrificed after a 12-day treatment course and the tumors were harvested to be photographed. The investigators measuring tumor volumes were blinded to the grouping information.

**Gene knockdown and overexpression**

Sequences of shRNAs were shown in Table S1 and cloned into Tet-pLKO-puro, whereas coding genes of luciferase (Luc), FLAG-PDCD11 (1-1032), or His-NLS-PDCD11 (1033-1431) were cloned into pLVX-TetOne-Puro. Lentivirus packaging and transduction were performed according to our previous work [1-3]. After selection using 2 µg/mL puromycin, cells were treated with 100 ng/mL Doxy for 5 days to induce expression of shLuc/shPDCD11, or with 1 µg/mL Doxy for 2 days to induce overexpression of Luc, PDCD11 (1-1032), or PDCD11 (1033-1431).

Downregulation of RSL1D1 in HCT116*^p53+/+^* cells was achieved using a pair of previously verified siRNAs (Table S1) [1]. 48 h post transfection of siNC/siRSL1D1, cells were harvested for western blotting. To achieve overexpression of PDCD11, pBiFC-mCherreyN159-FLAG or pBiFC-mCherryN159-FLAG-PDCD11 were transfected into HCT116*^p53+/+^* cells using lipo2000. 24 h post transfection, the cells with various treatments were harvested for different assays. To achieve transient gene overexpression in the cells expressing shPDCD11, pcDNA3 plasmids were transfected into the cells already treated with 100 ng/mL Doxy for 2 days, followed by continuous incubation with Doxy. 24 h later, cells were dissociated and seeded into new dishes. 72 h post transfection, cells were harvested for western blotting.

**qRT-PCR and western blot analyses**

qRT-PCR and western blot analyses were performed according to our previous work [1, 3, 4]. All the primers for qRT-PCR were shown in Table S1. Primary antibodies used for western blotting in this study were against PDCD11 (homemade mouse monoclonal), GAPDH (rabbit polyclonal #abs130609, Absin), FLAG (mouse monoclonal M2, Sigma-Aldrich), p53 (mouse monoclonal DO-1, Santa Cruz), HDM2 (rabbit monoclonal D1V2Z, CST), p21 (rabbit monoclonal 12D1, CST), Ubiquitin (mouse monoclonal P4D1, CST), CDC25C (rabbit monoclonal #A12234, Abclonal), CDK1 (rabbit monoclonal #A11420, Abclonal), p-CDK1-Y15 (rabbit polyclonal #AP0016, Abclonal), Caspase-3 (rabbit polyclonal #A2156, Abclonal), His tag (mouse monoclonal #K200060M, Solarbio), p14ARF (rabbit monoclonal #AG1582, Beyotime), and S-tag (mouse monoclonal #AF0285, Beyotime).

**Cell growth and cell cycle**

Cell growth curves were drawn by counting cells after different culture times. Cell cycle was investigated by performing PI assay according to our previous work [1, 3].

**Immunoprecipitation and co-immunoprecipitation assays**

Immunoprecipitation (IP) assay was performed to investigate p53 ubiquitination. Lysate supernatant of the cells treated with MG-132 (20 µM, 8 h) were incubated with anti-p53 (DO-1) and Protein A+G magnetic beads successively. The beads were boiled in 1 × SDS-PAGE loading buffer for western blot analysis. Anti-p53 (DO-1) and anti-ubiquitin (P4D1) were used to determine the levels of non-ubiquitinated and ubiquitinated p53, respectively. HRP-labeled secondary antibody against mouse IgG light chain (#A25012, Abbkine) was used to exclude interference from antibody heavy chains.

Co-IP assay was performed to verify the endogenous PDCD11-p53 and PDCD11-HDM2 interactions *in vivo*. Anti-p53 (DO-1) and anti-HDM2 (D1V2Z) were used to immunoprecipitate p53 and HDM2, respectively.

**GST pulldown assay**

GST pulldown assay was performed to investigate protein-protein interaction *in vitro* according to our previous work [1, 2]. Coding genes of PDCD11 and its truncated variants were cloned into pET-32a(+), whereas coding genes of p53, HDM2, and their truncations were cloned into pGEX-6P-1 or pGEX-5X-1. These vectors were transformed into *E.coli* BL21(DE3) for IPTG-induced expression of target genes. The recombinant proteins with His-S or GST tags were purified by affinity chromatography.

**Immunofluorescence, proximity ligation, and bimolecular fluorescence complementation assays**

Immunofluorescence (IF) assay was performed to investigate the subcellular localization of proteins according to our previous work [1]. Primary antibodies used for IF assay were against PDCD11 (homemade), FLAG (M2), p53 (rabbit monoclonal 7F5, CST), HDM2 (D1V2Z), and His tag (#K200060M, Solarbio).

Proximity ligation assay (PLA) was performed to investigate endogenous protein-protein interaction *in vivo* using Duolink® In Situ Orange Starter Kit (#DUO92102, Sigma-Aldrich) as per the kit manual. Primary antibody pairs used in this assay included anti-PDCD11 (homemade)/anti-p53 (7F5), anti-PDCD11 (homemade)/anti-HDM2 (D1V2Z), anti-FLAG (M2)/anti-p53 (7F5), anti-His (#K200060M, Solarbio)/anti-HDM2 (D1V2Z), or anti-p53 (DO-1)/anti-HDM2 (D1V2Z). Incubation with a primary antibody pair of mouse/rabbit IgGs was used as a negative control.

Bimolecular fluorescence complementation (BiFC) assay was another method to investigate protein-protein interaction *in vivo* in this study. Cells were co-transfected with previously verified plasmid pairs of pBiFC-mCherryN159/mCherryC160 [1, 2]. The coding region of PDCD11 was cloned into pBiFC-mCherryN159, whereas the coding regions of p53 and HDM2 were cloned into pBiFC-mCherryC160. 24 h after transfection, the BiFC fluorescence in the cells can be observed. Co-transfection with an empty plasmid pair was used as a negative control.

To investigate the colocalization of PDCD11, p53, and HDM2 proteins, a previously verified combinational BiFC-IF assay [1, 2] was performed. Cells co-transfected with pBiFC-mCherryN159-PDCD11 and pBiFC-mCherryC160-p53/HDM2 pairs were subjected to IF assay using anti-HDM2 (D1V2Z)/anti-p53 (7F5).

For all the assays, cell nuclei were stained using Hoechst33258, prior to be observed under a laser confocal microscope.

**Statistical analysis**

All numerical data are shown as mean ± SD. Significance of the difference between two groups was determined using two-tailed Student’s t-test. **P <* 0.05; ***P <* 0.01 denote significant difference. The variance similar between the groups was statistically compared using F-test.

**References**

1. Ding L, Zhang Z, Zhao C, Chen L, Chen Z, Zhang J, et al. Ribosomal L1 domain-containing protein 1 coordinates with HDM2 to negatively regulate p53 in human colorectal Cancer cells. J Exp Clin Cancer Res. 2021;40:245.

2. Ding L, Zhao C, Xu Y, Zhang Z, Nie Y, Liao K, et al. Mutations in DNA binding domain of p53 impede RSL1D1-p53 interaction to escape from degradation in human colorectal cancer cells. Exp Cell Res. 2022;417:113211.

3. Zhang X, He Y, Lee KH, Dubois W, Li Z, Wu X, et al. Rap2b, a novel p53 target, regulates p53-mediated pro-survival function. Cell Cycle. 2013;12:1279-1291.

4. Ding L, Gao Q, Xu Z, Cai L, Chen S, Zhang X, et al. An Inter-Supplementary Biohybrid System Based on Natural Killer Cells for the Combinational Immunotherapy and Virotherapy of Cancer. Adv Sci (Weinh). 2022;9:e2103470.

**Table S1 Oligonucleotides in this study.**

| Primers for qRT-PCR | Sequences (5’-3’) |
| --- | --- |
| GAPDH_Fwd | TGGGCTACACTGAGCACCAG |
| GAPDH_Rev | GGGTGTCGCTGTTGAAGTCA |
| PDCD11_Fwd | TTTGCCCAGCTTGAGTTTCAG |
| PDCD11_Rev | TGTCGATATAGACCGACCAGACA |
| p53_Fwd | CCAGAAAACCTACCAGGGCA |
| p53_Rev | GAATGCAAGAAGCCCAGACG |
| HDM2_Fwd | CCGGATCTTGATGCTGGTGT |
| HDM2_Rev | CTGATCCAACCAATCACCTGAAT |
| p21_Fwd | AGCGATGGAACTTCGACTTTG |
| p21_Rev | CGAAGTCACCCTCCAGTGGT |
| CDC25C_Fwd | GCATTTAGCTGGGATGAATCATG |
| CDC25C_Rev  CDK1_Fwd  CDK1_Rev | AAGAAGCTGTGCTGGGCTACA  CCTCAAAATCTCTTGATTGATGACA  GCTCTGGCAAGGCCAAAAT |
| shRNA/siRNA | Sequences (5’-3’) |
| Upper_shLuc | CCGGCGCTGAGTACTTCGAAATGTCCTCGAGGACATTTCGAAGTACTCAGCGTTTTT |
| Bottom_shLuc | AATTAAAAACGCTGAGTACTTCGAAATGTCCTCGAGGACATTTCGAAGTACTCAGCG |
| Upper_shPDCD11 | CCGGGCAGTCAGTTGAACAAGACAACTCGAGTTGTCTTGTTCAACTGACTGCTTTTTG |
| Bottom_shPDCD11  Sense_siNC  Antisense_siNC  Sense_siRSL1D1  Antisense_siRSL1D1 | AATTCAAAAAGCAGTCAGTTGAACAAGACAACTCGAGTTGTCTTGTTCAACTGACTGC  UUCUCCGAACGUGUCACGUTT  ACGUGACACGUUCGGAGAATT  CGAAGGAUGAACCCAAUUCAATT  UUGAAUUGGGUUCAUCCUUCGTT |

**Figure S1**


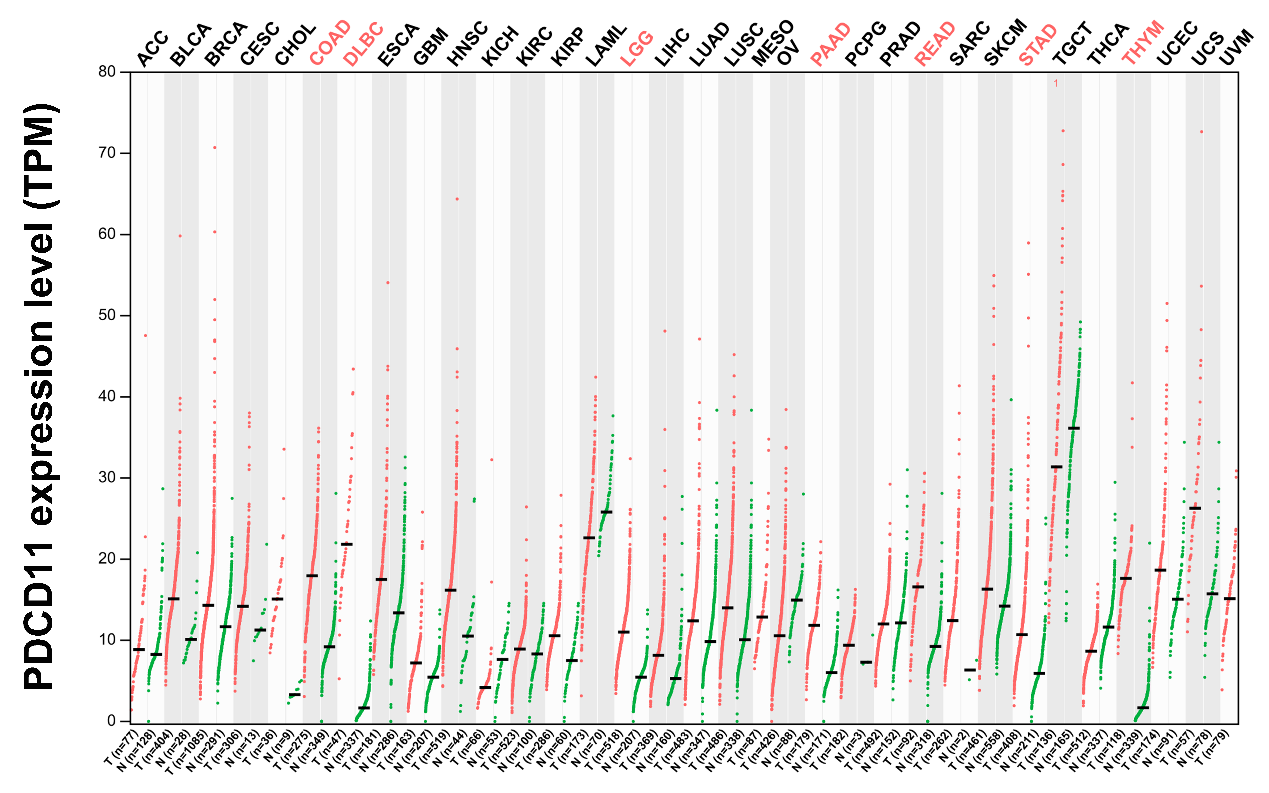


**Fig. S1 Expression profiles of PDCD11 in different cancer types.** Red marked cancer types possess a higher PDCD11 expression level in tumor than in normal tissues. Data from TCGA and GTEx databases were analyzed using GEPIA2. FC ≥ 1.5 and q-value < 0.001 denote significant difference.

**Figure S2**


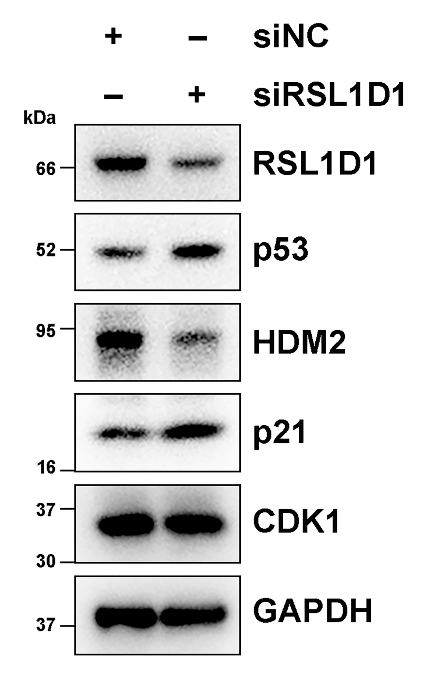


**Fig. S2 Downregulation of RSL1D1 does not affect the level of CDK1 protein.** Western blot analyses were performed to determine the levels of RSL1D1, p53, HDM2, p21, and CDK1 proteins in HCT116 cells transfected with siNC or siRSL1D1. GAPDH was used as a loading control.

**Figure S3**


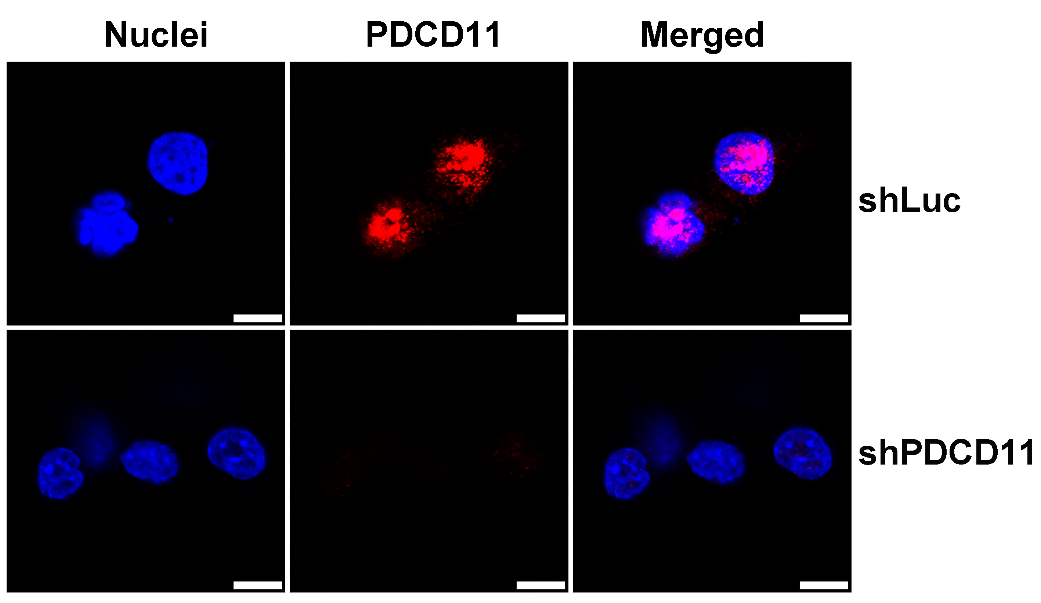


**Fig. S3 Homemade anti-PDCD11 is suitable for IF assay.** All the lentivirus-transduced cells were treated with Doxy to induce expression of shRNAs. Homemade anti-PDCD11 was used for IF assay to stain PDCD11 (red) in HCT116 cells and the nuclei were stained to blue. Bars: 10 μm.

**Figure S4**


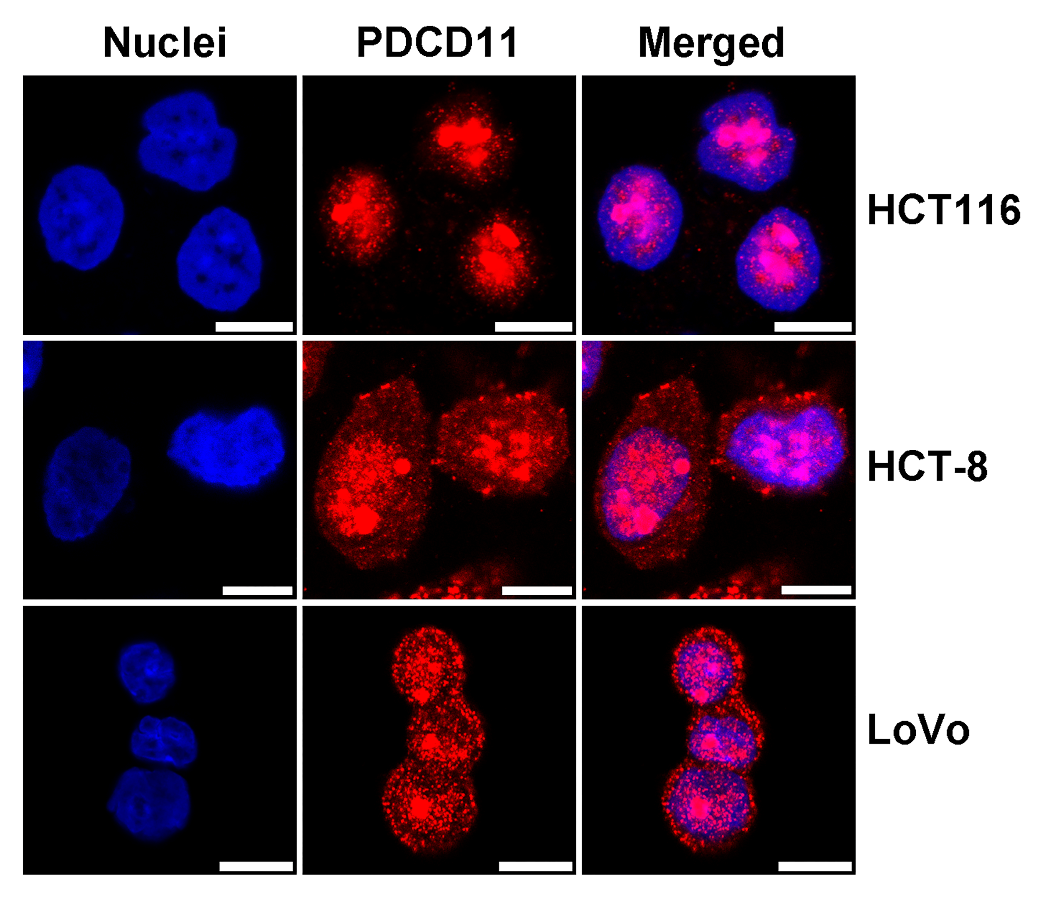


**Fig. S4 PDCD11 shows an “Extra-nucleolar” distribution in a panel of colorectal cancer cells harboring wild-type p53.** IF assay was performed to investigate the subcellular localization of PDCD11. The nuclei were stained to blue. Scale bars: 10 μm.

**Figure S5**


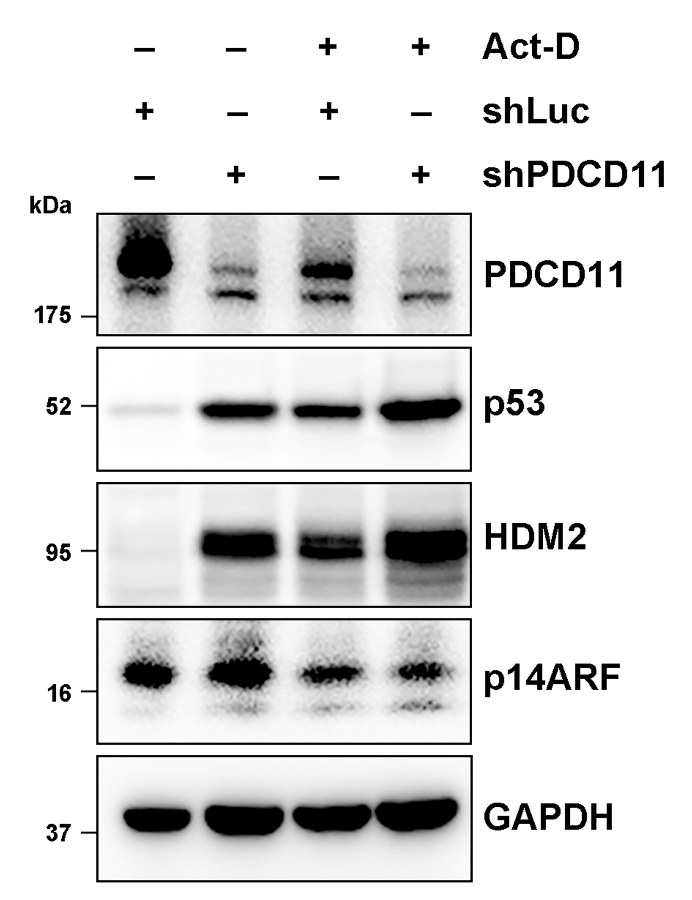


**Fig. S5 PDCD11 negatively regulates p53 under either normal condition or nucleolar stress.** All the lentivirus-transduced cells were treated with Doxy to induce expression of shRNAs. Western blot analyses were performed to determine the levels of PDCD11, p53, HDM2, and p14ARF proteins in HCT116 cells with or without treatment of Act-D (5 nM, 24 h). GAPDH was used as a loading control.

**Figure S6**


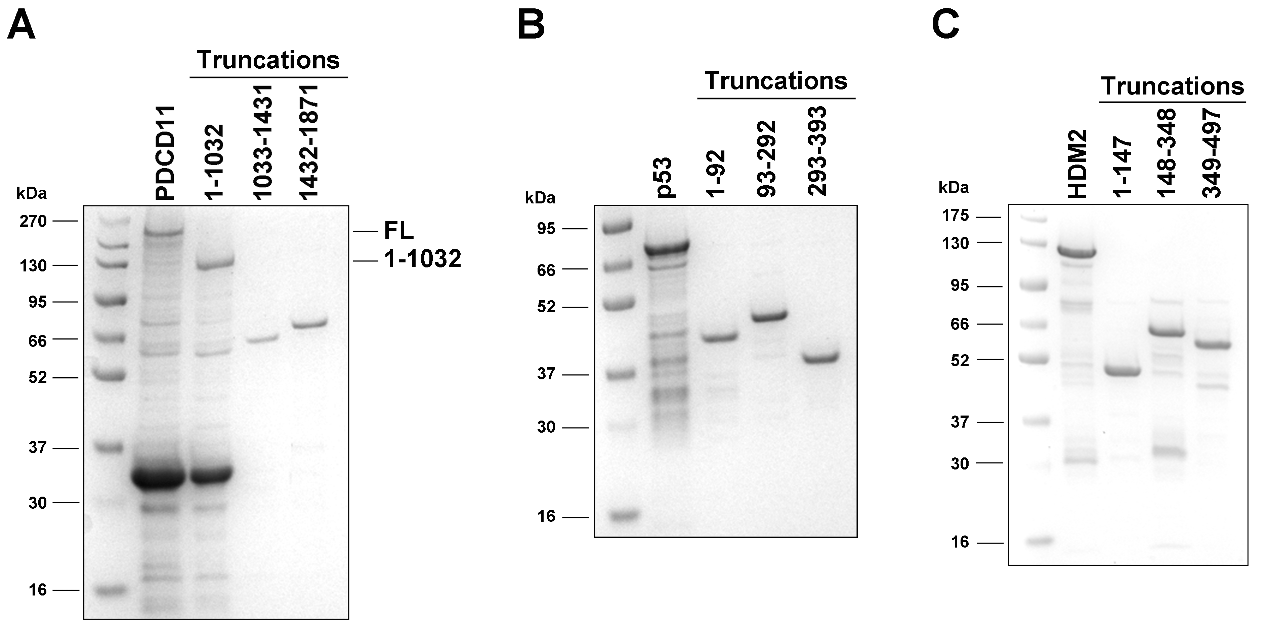


**Fig. S6 SDS-PAGE analyses of purified recombinant proteins. A** PDCD11-FL and its truncations (aa 1-1032, aa 1033-1431, and aa 1432-1871) were expressed in prokaryotic cells and purified by affinity chromatography. The purified His-S-tagged proteins were subjected to SDS-PAGE analysis. **B** p53-FL and its truncations (aa 1-92, aa 93-292, and aa 293-393) were expressed in prokaryotic cells and purified by affinity chromatography. The purified GST-tagged proteins were subjected to SDS-PAGE analysis. **C** HDM2-FL and its truncations (aa 1-147, aa 148-348, and aa 349-497) were expressed in prokaryotic cells and purified by affinity chromatography. The purified GST-tagged proteins were subjected to SDS-PAGE analysis.

**Figure S7**


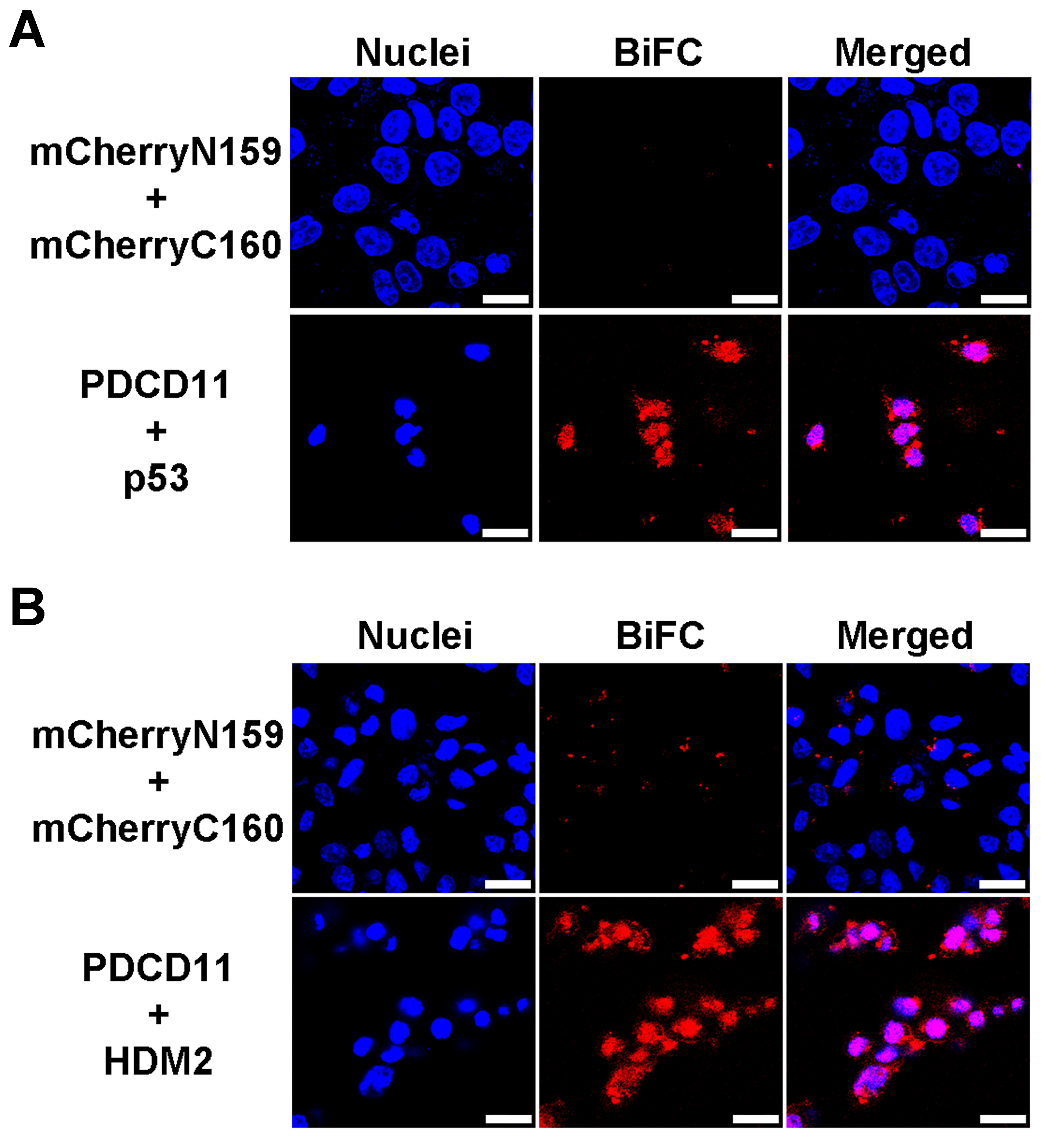


**Fig. S7 BiFC analyses of PDCD11-p53 and PDCD11-HDM2 interactions in HCT116 cells.** BiFC assay was performed to investigate the interaction between PDCD11, p53, and HDM2 *in vivo*. **A** HCT116*^p53-/-^* cells were co-transfected with pBiFC-mCherryN159-PDCD11 and pBiFC-mCherryC160-p53. **B** HCT116*^p53+/+^* cells were co-transfected with pBiFC-mCherryN159-PDCD11 and pBiFC-mCherryC160-HDM2. **A** and **B** The interaction between two proteins fused with mCherryN159 and mCherryC160, respectively, was indicated by the red fluorescence in the cells. Co-transfection with an empty plasmid pair was used as a negative control. The nuclei were stained to blue. Scale bars: 20 μm.

**Figure S8**


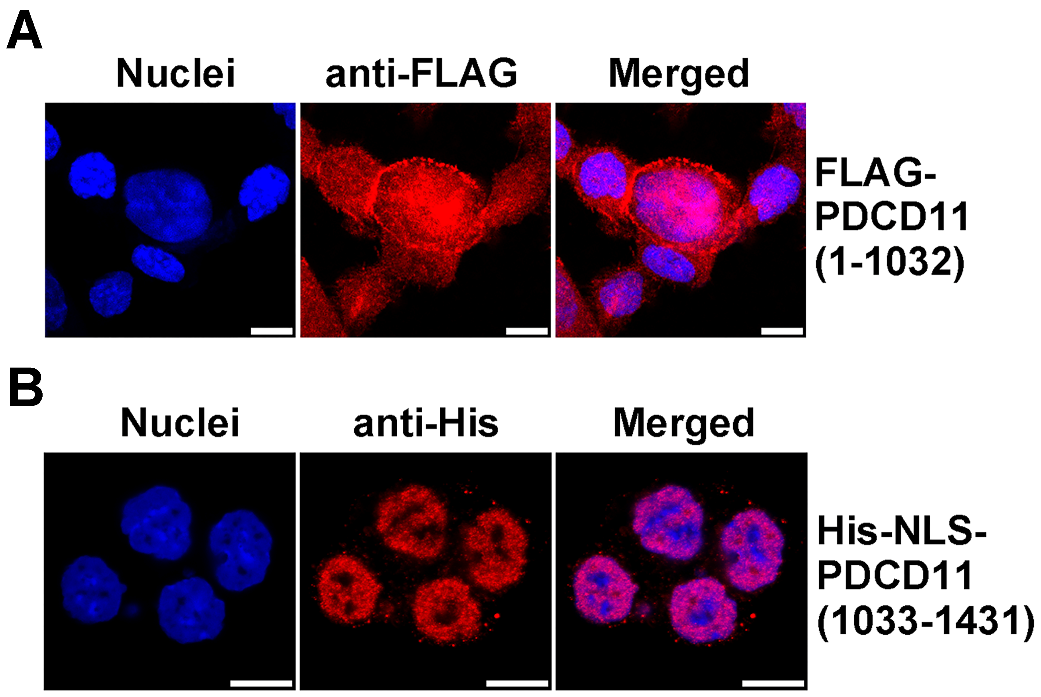


**Fig. S8 Subcellular localization of truncated PDCD11.** Lentivirus-transduced HCT116 cells were treated with Doxy to induce overexpression of truncated PDCD11. **A** IF assay was performed to investigate the subcellular localization of FLAG-PDCD11 (1-1032) (red) using anti-FLAG. **B** IF assay was performed to investigate the subcellular localization of His-NLS-PDCD11 (1033-1431) (red) using anti-His tag. **A** and **B** The nuclei were stained to blue. Scale bars: 10 μm.

**Figure S9**


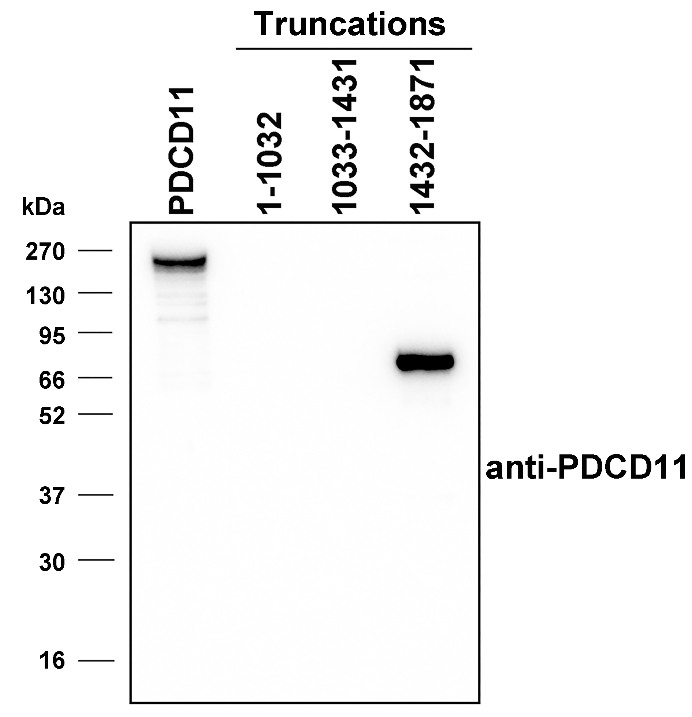


**Fig. S9 Homemade anti-PDCD11 specifically recognizes aa 1432-1871 of PDCD11 protein.** Western blotting was performed to identify the epitope recognized by the homemade monoclonal antibody against PDCD11. Purified His-S-tagged PDCD11 (FL, aa 1-1032, aa 1033-1431, and aa 1432-1871) were loaded onto the gel for blotting.
